# Definitive Cefazolin Treatment for Community-Onset Enterobacteriaceae Bacteremia Based on the Contemporary CLSI Breakpoint: Clinical Experience of a Medical Center in Southern Taiwan

**DOI:** 10.3390/antibiotics8040216

**Published:** 2019-11-10

**Authors:** Ching-Chi Lee, Chung-Hsun Lee, Po-Lin Chen, Chih-Chia Hsieh, Hung-Jen Tang, Wen-Chien Ko

**Affiliations:** 1 Graduate Institute of Medical Sciences, College of Health Sciences, Chang Jung Christian University, Tainan 71101, Taiwan; chichingbm85@gmail.com; 2 Department of Adult Critical Care Medicine, Tainan Sin-Lau Hospital, Tainan 70142, Taiwan; 3 Department of Emergency Medicine, National Cheng Kung University Hospital, College of Medicine, National Cheng Kung University, Tainan 701, Taiwan; chlee82er@yahoo.com.tw (C.-H.L.); hsiehchihchia@gmail.com (C.-C.H.); 4 Department of Medicine, College of Medicine, National Cheng Kung University, Tainan 701, Taiwan; cplin@mail.ncku.edu.tw; 5 Department of Internal Medicine, National Cheng Kung University Hospital, College of Medicine, National Cheng Kung University, Tainan 701, Taiwan; 6 Department of Medicine, Chi-Mei Medical Center, Tainan 71004, Taiwan; 7 Department of Health and Nutrition, Chia Nan University of Pharmacy and Science, Tainan 71710, Taiwan

**Keywords:** cefazolin, definitive therapy, community, bacteremia, *Escherichia coli*, *Klebsiella pneumoniae*, *Proteus mirabilis*

## Abstract

Cefazolin is traditionally active against Escherichia coli, Klebsiella species, and Proteus mirabilis (EKP) isolates. The Clinical and Laboratory Standards Institute (CLSI) has twice updated cefazolin susceptibility breakpoints for EKP since 2010, but its role in the definitive treatment of cefazolin-susceptible EKP bacteremia remains debated. To assess its efficacy as a definitive agent, the 8-year cohort study consisted of 941 adults with monomicrobial cefazolin-susceptible EKP bacteremia, based on the CLSI criteria issued in 2019, was retrospectively established in a medical center. Based on the definitive antimicrobial prescription, eligible patients were categorized into the cefazolin (399 patients, 42.4%) and broader-spectrum antibiotic (BSA) (542, 57.6%) groups. Initially, fewer proportions of patients with fatal comorbidities (the McCabe classification) and the critical illness (a Pitt bacteremia score ≥4) at the onset and day 3 of the bacteremia episode were found in the cefazolin group, compared to the BSA group. After propensity-score matching, no significant difference of patient proportions between the cefazolin (345 patients) and BSA (345) groups was observed, in terms of the elderly, types and severity of comorbidities, bacteremia severity at the onset and day 3, major bacteremia sources, and the 15-day and 30-day crude mortality. In early outcomes, lengths of time to defervescence, intravenous (IV) antimicrobial administration, and hospitalization were similar in the two matched groups; lower costs of IV antimicrobial administration were observed in the cefazolin group. Notably, for late outcomes, lower proportions of post-treatment infections caused by antimicrobial-resistant pathogens (ARPs) and post-treatment mortality rates were evidenced in the cefazolin group. Conclusively, cefazolin is definitively efficacious and cost-effective for adults with community-onset cefazolin-susceptible EKP bacteremia in this one-center study, compared to BSAs. However, a prospective multicenter study should be conducted for external validation with other communities.

## 1. Introduction

Cefazolin, a parenteral first-generation cephalosporin (GC) available for study in 1972, has in vitro bactericidal activity against staphylococci, streptococci, *Escherichia coli*, *Klebsiella pneumoniae*, and *Proteus mirabilis* [1], and gives sustained antibacterial concentrations in blood after intravenous or intramuscular use [2]. However, numerous investigations have only studied its efficacy in surgical prophylaxis [3] and varied community-acquired Gram-positive infections, such as skin and soft-tissue infections [4], bone and joint infections [5], and continuous ambulatory peritoneal dialysis infections [6]. Although the CLSI has twice updated cefazolin susceptibility breakpoints for EKP isolates since 2010 [7,8], a clinical report of the therapeutic role of definitive cefazolin evidenced for bloodstream infections is lacking. Therefore, within the scope of community-onset bacteremia caused by cefazolin-susceptible EKP isolates, based on the contemporary susceptibility standard, we compared efficacies of definitive cefazolin treatment with other definitive agents that have a broader spectrum.

## 2. Materials and Methods

### 2.1. Study Design and Population

The study institution was a medical center, a university-affiliated hospital located in Tainan, a metropolitan city in southern Taiwan. Of adults with blood cultures sampled in the emergency department (ED) during the period between January 2007 and December 2014, growth in blood cultures was retrospectively screened in a database of electric chart records. Adults with monomicrobial EKP bacteremia were initially included. In cases with multiple bacteremic episodes, only the first episode was included for each patient. Patients were excluded if they were transferred from other hospitals, were diagnosed with bloodstream infections before ED arrival, received inadequate empirical therapy, had not been hospitalized through the ED, had a fatality within 3 days after the bacteremia onset (i.e., patients not received definitive antibiotic therapy), had been infected by cefazolin non-susceptible EKP, or did not participate in the follow-up visit within the study endpoint. Finally, the cohort only consisted of adults with community-onset monomicrobial bacteremia caused by cefazolin-susceptible EKP isolates and received appropriate empirical antimicrobial therapy. The study was approved by the institutional review board of National Cheng Kung University Hospital (B-ER-106-294), and the requirement of obtaining informed consent was waived.

### 2.2. Data Collection

Clinical variables were retrospectively collected by reviewing the medical records of all eligible patients by using a predetermined form, including patient demographics, vital signs and laboratory data at the ED, comorbidity types, comorbidity severity (the McCabe classification), the duration, dosage, and type of antimicrobial agents administered, bacteremia sources, bacteremia severity (a Pitt bacteremia score), the length of hospital stay, and patient fatality. To accurately collect the aforementioned information, the medical records were independently reviewed by two authors, and any discrepancy was solved by discussion between the authors. Based on definitive antimicrobials, eligible patients were categorized into those definitively treated with cefazolin (the cefazolin group); and those treated with definitive antimicrobials with the broader-spectrum than cefazolin (the BSA group), in terms of second-GCs, third-GCs, fourth-GCs, fluoroquinolones, carbapenems, ureidopenicillins, aminopenicillins/β-lactamase inhibitors, and ureidopenicillins/β-lactamase inhibitors.

The primary and secondary endpoint was 30-day crude mortality after the bacteremia onset and post-treatment crude mortality within 60 days after the end of IV antimicrobial therapy, respectively. The time-to-defervescence, lengths of IV antimicrobial administration and hospitalization, and costs of antimicrobial therapy were regarded as the indicator of early outcomes in the response to the definitive therapy. The post-treatment ARP infections, current bacteremia, and post-treatment crude mortality were included as variables to reveal the late outcomes in response to the definitive therapy. Early and late outcomes were evaluated at the 4- to 30-day follow-up visit after the bacteremia onset and within 60 days after the end of IV antimicrobial therapy, respectively.

### 2.3. Definitions

The episode of bacteremia in the community was diagnosed as community-onset bacteremia [9,10]. Because the susceptibility data was available approximately at day 3 of the bacteremia episode, empirical antibiotic therapy was defined as the drug prescribed within three days after the bacteremia onset, whereas definitive therapy was referred to the antibiotic prescribed when the susceptibility result was available. As previously described [9,10], the antibiotic therapy was considered appropriate if all of the following criteria were fulfilled: (i) the route and dosage was administered in accordance of recommendations in the Sanford Guide [11]; (ii) causative microorganisms were in vitro susceptible to administrated antibiotics according to the contemporary CLSI breakpoint [8]. The period between the bacteremia onset (i.e., ED arrival) and administration of appropriate antimicrobials was regarded as the time-to-appropriate antibiotic and inappropriate empirical therapy was defined as the time-to-appropriate antibiotic of >24 h [10,12].

A Pitt bacteremia score [10,13], a previously validated scoring system based on vital signs, usage of vasopressor agents, mental status, receipt of mechanical ventilation and recent cardiac arrest, was utilized to assess the bacteremia severity at the bactereamia onset (i.e., ED arrival). To further grade the bacteremia severity as an initiation of definitive antibiotic therapy, a Pitt bacteremia score at day 3 of the bacteremia episode was evaluated; patients became stabilized at day 3, as indicated by a Pitt bacteremia score = 0, and those remained critically ill, as indicated by a Pitt bacteremia score ≥ 4.

As previously indicated [14], the ARP was defined as a microorganism with broader-spectrum resistance than that of the same isolate in the community; for instance, these microorganisms could be Gram-negative pathogens (e.g., Enterobacteriaceae, *Pseudomonas* species, *Vibrio* species, or *Aeromonas* species) resistant to extended-spectrum cephalosporins or fluoroquinolones, methicillin-resistant *Staphylococcus aureus*, ampicillin-resistant enterococci, and penicillin-resistant streptococci. Defervescence, as previously described [15], was defined as an afebrile state in which the body temperature was maintained at <37.0 °C for at least 24 h, and time-to-defervescence was defined as the period between defervescence and administration of appropriate antimicrobials. Recurrent bacteremia was defined as a new episode of the documented bloodstream infection caused by the same microorganism and in vitro susceptibility as the index bacteremia episode. Comorbidities were defined as previous descriptions [16] and the comorbid severity was assessed by a previously established McCabe classification [17]. Like previous definitions [18], the removal of infected hardware, drainage of infected fluid collections, or resolution of obstruction for biliary or urinary sources was referred as appropriate control of bacteremia source. Crude mortality was used to define death from all causes.

### 2.4. Microbiological Methods

EKP isolates were prospectively stored in the study hospital and identified by a Gram-Negative-Identification Card of the Vitek 2 system (bioMe’rieux, Lyon, France). Antimicrobial susceptibilities were determined by the disk diffusion method, based on contemporary CLSI standards [8]. For cefazolin, susceptibility breakpoints were set at susceptible (≥23 mm), intermediate (20–22 mm), and resistant (≤19 mm). Other tested drugs included levofloxacin, cefazolin, cefuroxime, cefotaxime, ceftazidime, cefepime, ertapenem, ampicillin/sulbactam, piperacillin, and piperacillin/tazobactam. If patient empirically or definitively treated by other agents, the susceptibility of the indicated agent was measured.

### 2.5. Statistical Analysis

The Statistical Package for the Social Science for Windows (SPSS, Chicago, IL, USA, version 23.0) was applied for the statistical analysis. Continuous and categorical variables were compared by the Student’s *t* test and the *Chi*-square or Fisher’s exact test, respectively. To identify the independent determinants linked to 30-day crude mortality, all variables of 30-day crude mortality with *P* values less than 0.05 in the univariate analysis were conducted for the stepwise, backward logistic regression model. A *P* value less than 0.05 was considered significant.

A propensity-score (PS) matched analysis was performed to control for confounding variables in the choice of definitive antimicrobials. The PS was calculated by the independent determinants of 30-day crude mortality. Patients in the cefazolin group were matched at a ratio of 1:1 with those in the BSA group, using individual PSs. The matching by the closest total PS was done manually based on a tolerance interval approach with the PS difference of 0.2 [19].

## 3. Results

### 3.1. Demographics and Clinical Characteristics of the Entire Cohort

**Figure 1:**
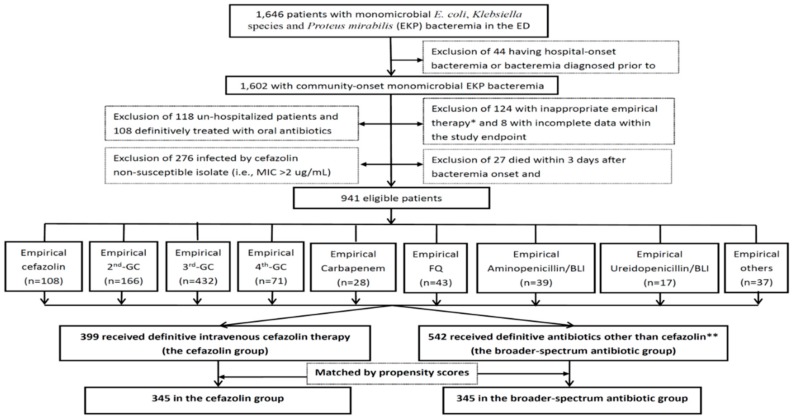
Flowchart of patient selections. * Indicate the time to appropriate antibiotic of >24 h. ** Included 235 patients with third-GCs, 154 with second-GCs, 24 with carbapenems, 60 with FQs, 30 with fourth-GCs, 21 with ampicillin/sulbactam, 13 with piperacillin/tazobactam, 3 with piperacillin, and 2 with others. ED = emergency department; GC = generation cephalosporin; FQ = fluoroquinolone; BLI = β-lactamase inhibitor.

A total of 941 adults were included based on the inclusion and exclusion criteria (Figure 1) and categorized into the cefazolin (399 patients, 42.4%) and BSA groups (542, 57.6%). Of the total 941 patients, their mean age was 67.8 years, and 545 (57.9%) were female. The leading comorbidities included hypertension (462 patients, 49.1%), diabetes mellitus (380, 40.4%), malignancies (251, 26.7%), neurological diseases (195, 20.7%), liver cirrhosis (126, 13.4%), chronic kidney diseases (122, 13.0%), and coronary artery diseases (88, 9.4%). Common sources of bacteremia were urinary tract infections (476 patients, 50.6%), intra-abdominal infections (118, 12.5%), pneumonia (91, 9.7%), biliary tract infections (88, 9.4%), liver abscess (73, 7.8%), primary bacteremia (64, 6.8%), and skin and soft-tissue infections (22, 2.3%).

The median (interquartile range) length of ED stay and hospital stay was 16.2 (6.0–26.4) hours and 10 (7–15) days, respectively. The proportion of critically ill patients at the onset and day 3 of the bacteremia episode was 15.6% (147 patients) and 11.3% (106), respectively. The 15-day and 30-day crude mortality rate was 3.2% (30 patients) and 6.6% (62), respectively.

### 3.2. Comparisons of Baseline Characteristics and Severity between Two Groups

Univariate analyses were used to compare two patient groups (Table 1). The higher proportion of females, bacteremia due to urinary tract infections and a low Pitt bacteremia score (=0) at day 3 as well as the lower 15-day or 30-day crude mortality rate were present in the cefazolin group. Otherwise, less patients of nursing-home residents, comorbid malignancies, fatal comorbidities (the McCabe classification), bacteremia due to intra-abdominal infections or pneumonia, and a high Pitt bacteremia score (≥4) at the bacteremia onset or day 3 were disclosed in the cefazolin group.

### 3.3. Risk Factors of 30-Day Crude Mortality in the Overall Cohort

For the entire cohort, the association of clinical variables with 30-day mortality, in terms of old age, gender, bacteremia severity at the onset and day 3, major sources of bacteremia, comorbidity severity, major comorbidities, and major causative pathogens, was examined by the univariate analysis (Table 2). The following variables were positively associated with 30-day mortality: male patients, a high Pitt bacteremia score (≥4) at the onset, bacteremic pneumonia, fatal comorbidities (the McCabe classification), and comorbid malignancies or liver cirrhosis. Additionally, a low Pitt bacteremia score (=0) at day 3, bacteremia due to urinary tract infections, and comorbid hypertension was negatively associated with 30-day mortality. In the multivariate regression, only seven independent determinants were identified: a high Pitt bacteremia score (≥4) at the onset, a low Pitt bacteremia score (=0) at day 3, fatal comorbidities, underlying malignancies or liver cirrhosis, and bacteremia because of pneumonia or urinary tract infections.

### 3.4. Baseline Characteristics, Severity, and Outcomes in PS-Matched Groups

Out of 399 patients who underwent definitive cefazolin therapy, 345 were matched with 345 in the BSA group. After appropriate matching (Table 1), no significant differences in the patient proportion between two groups were observed, in terms of the elderly, comorbidity types, severity of comorbidities, bacteremia severity at the onset and day 3, and major bacteremia sources. Dissimilar proportions were only observed in gender patients and nursing-home residents. More importantly, the 15-day and 30-day crude mortality rate did not differ in the two groups.

**Figure 2:**
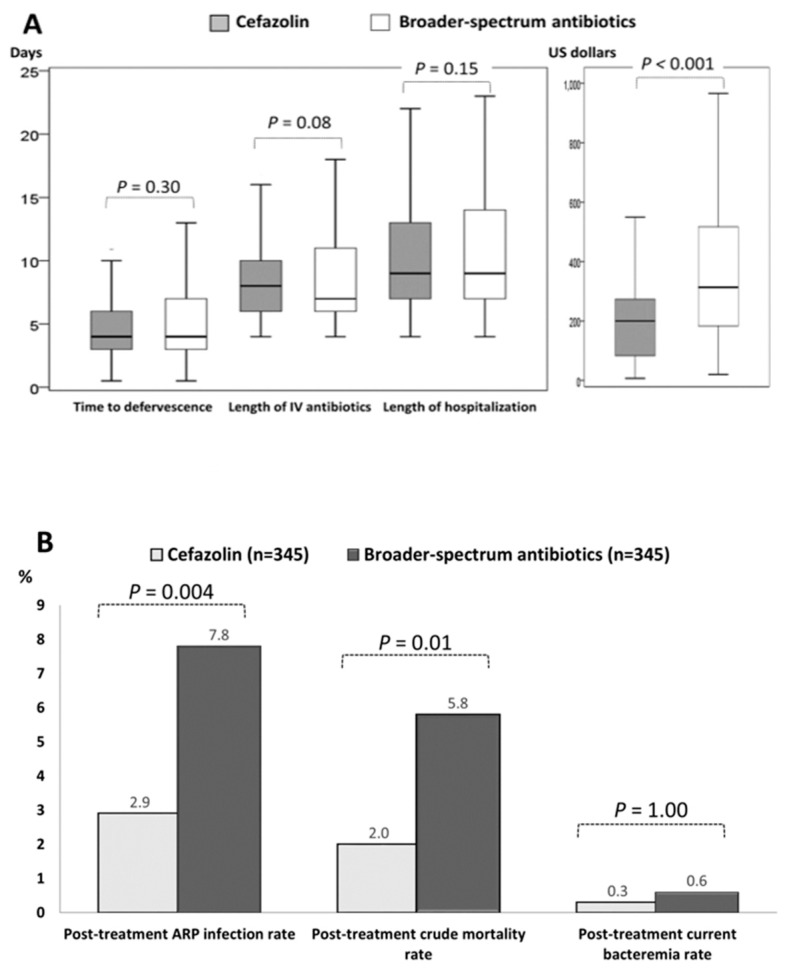
Boxplots for early (**A**) and late (**B**) outcomes in response to definitive therapy in the matched cohort. ARP = antimicrobial-resistant pathogen; IV= intravenous.

Early and late outcomes in response to definitive therapy in the two matched groups were exhibited in Figure 2. For early outcomes (Figure 2A), in terms of the time-to-defervescence and lengths of IV antimicrobial therapy and hospital stay, were similar between the two groups. Notably, only the lower cost of IV antimicrobial administration was observed in the cefazolin group. Of importance, focusing on late outcomes indicative of the adverse events within 60 days after the end of IV antimicrobial therapy (Figure 2B), despite the similar proportion of post-treatment current bacteremia between two groups, lower proportions of post-treatment ARP infections (2.9% vs. 7.8%, *p* = 0.004) and post-treatment crude mortality rates (2.0% vs. 5.8%, *p* = 0.01) were evidenced in the cefazolin group, compared to the BSA group.

## 4. Discussion

Although the in vitro activity of cefazolin against EKP isolates is disclosed in the community [1] and it offers sustained antibacterial serum concentrations after intravenous use [2], clinical information on the treatment of bloodstream infections (except urospesis) is limited [20]. However, because of its narrow spectrum, administration of IV cefazolin as the empirical agent is inadequate. Therefore, to highlight the therapeutic role of cefazolin for the definitive treatment of EKP bacteremia, the long-term cohort study was conducted herein. Although the baseline characteristic at initial administration of definitive antimicrobials, particularly in severity of bacteremia and comorbidities, differed between the cefazolin and BSA groups, we used PS matching to overcome these vast differences. Consequently, the present study revealed that definitive cefazolin therapy was safer and more cost-effective than BSA therapy, if the patient was stabilized after 72 h of appropriate empirical antimicrobial therapy.

Cefazolin interpretive criteria for EKP isolates have been revised twice by the CLSI since 2010. In 2010 and 2012, the CLSI released recommended susceptible breakpoints for cefazolin (MICs ≤1 mg/L and ≤2 mg/L, respectively). However, such an alteration has not been specifically evidenced for patients having bloodstream infections. Herein, our cohort presented the successful effect of definitive cefazolin therapy for community-onset bacteremia caused by “current cefazolin-susceptible” EKP isolates and thereby offered the clinic relevance supporting the updated CLSI standard.

This study has several limitations inherent in its design. First, a favorable clinical outcome in patients definitively treated by cefazolin should be cautiously interpreted, because our target population was limited to the less critically ill patients after appropriate empirical therapy and the study was conducted in a single tertiary hospital. A lack of an independent validation to other populations with varied bacteremia severity and sources is the leading of the study limitations. Second, this cohort study was retrospectively conducted to capture clinical information. To reduce recall bias, the medical records were independently reviewed by two authors. Third, the certain proportion (408/1602, 25.5%) of the entire cohort were excluded and such a bias of patient selection should be considered. Finally, although the patients who received cefazolin therapy through an appropriate route or dosage (1–2 g every 8 h, adjustments based on the renal function) were included, dissimilar efficacies of differential cefazolin dosage should be considered. Moreover, assessing the impacts of different antimicrobial classes on clinical outcomes would be difficult in the BSA group.

## 5. Conclusions

For adults with community-onset bacteremia caused by cefazolin-susceptible EKP, based on the contemporary CLSI criteria, and stabilized after 72 h of appropriate empirical therapy, definitive cefazolin therapy was safe and cost-effective during the eight years of experience in one medical center. However, a multicenter prospective study of the incorporation of definitive cefazolin therapy into antibiotic stewardship programs should be performed in the future for external validation with other communities.

## Figures and Tables

**Table 1:** Clinical characteristics and patient outcomes in the cefazolin and broader-spectrum antibiotic (BSA) groups.

Characteristics	Patient Numbers (%)					
	Overall Cohort			Matched Cohort		
	Cefazolin *n* = 399	BSA *n* = 542	*p* Value	Cefazolin *n* = 345	BSA *n* = 345	*p* Value
Gender, female	**278 (69.7)**	**267 (49.3)**	**<0.001**	**233 (67.5)**	**197 (57.1)**	**0.005**
The elderly, ≥65 years	244 (61.2)	321 (59.2)	0.55	210 (60.9)	201 (58.3)	0.49
Nursing-home residents	**3 (0.8)**	**16 (3.0)**	**0.02**	**2 (0.6)**	**10 (2.9)**	**0.02**
Time to antimicrobial switches, mean ± SD *	3.8 ± 1.2	3.8 ±1.7	0.69	3.9 ± 1.8	3.8 ± 2.3	0.36
Major comorbidities						
Hypertension	205 (51.4)	257 (47.4)	0.23	181 (52.5)	170 (49.3)	0.40
Diabetes mellitus	174 (43.6)	206 (38.0)	0.08	154 (44.6)	130 (37.7)	0.06
Malignancy	**87 (21.8)**	**164 (30.3)**	**0.004**	80 (23.2)	81 (23.5)	0.93
Neurological disease	80 (20.1)	115 (21.2)	0.66	72 (20.9)	67 (19.4)	0.64
Liver cirrhosis	50 (12.5)	76 (14.0)	0.51	47 (13.6)	44 (12.8)	0.74
Chronic kidney disease	42 (10.5)	80 (14.8)	0.06	37 (10.7)	53 (15.4)	0.07
Comorbidity severity (McCabe classification)			**0.001**			0.54
Ultimately and rapidly fatal	**63 (15.8)**	**133 (24.5)**		61 (17.7)	55 (15.9)	
Nonfatal	**336 (84.2)**	**409 (75.5)**		284 (82.3)	290 (84.1)	
Causative microorganisms						
***Escherichia coli***	**297 (74.4)**	**330 (60.9)**	**<0.001**	250 (72.5)	235 (68.1_	0.21
***Klebsiella* species**	**86 (21.6)**	**200 (36.9)**	**<0.001**	81 (23.5)	102 (29.6)	0.07
*Proteus mirabilis*	16 (4.0)	12 (2.2)	0.11	14 (4.1)	8 (2.3)	0.19
Major sources of bacteremia						
Urinary tract	**258 (64.7)**	**218 (40.2)**	**<0.001**	208 (60.3)	201 (58.3)	0.59
Biliary tract	33 (8.3)	55 (10.1)	0.33	31 (9.0)	33 (9.6)	0.79
Intra-abdominal	**37 (9.3)**	**81 (14.9)**	**0.009**	36 (10.4)	45 (13.0)	0.29
Primary bacteremia	27 (6.8)	37 (6.8)	0.97	26 (7.5)	21 (6.1)	0.45
Liver abscess	24 (6.0)	49 (9.0)	0.09	24 (7.0)	23 (6.7)	0.88
Pneumonia	**10 (2.5)**	**81 (41.9)**	**<0.001**	10 (2.9)	10 (2.9)	1.00
Inadequate source control during antibiotic therapy	12 (3.0)	25 (4.6)	0.21	10 (2.9)	11 (3.2)	0.83
Pitt bacteremia score						
at onset						
≥4	**40 (10.0)**	**107 (19.7)**	**<0.001**	33 (9.6)	24 (7.0)	0.21
0	128 (32.1)	143 (26.4)	0.06	114 (33.0)	109 (31.6)	0.68
at day 3						
≥4	**26 (6.5)**	**80 (14.8)**	**<0.001**	22 (6.4)	24 (7.0)	0.76
0	**332 (83.2)**	**369 (68.1)**	**<0.001**	286 (82.9)	291 (84.3)	0.61
Crude mortality rate						
15-day	**2 (0.5)**	**28 (5.2)**	**<0.001**	2 (0.6)	7 (2.0)	0.18
30-day	**9 (2.3)**	**53 (9.8)**	**<0.001**	9 (2.3)	15 (4.3)	0.21

SD = standard deviation. Data are given as number (percent), unless otherwise specified. Boldface indicates statistical significance, i.e., a *p* value of <0.05. * 296 patients without antimicrobial switch in the overall cohort and 224 in the matched cohort were respectively not calculated.

**Table 2:** Risk factors of 30-day crude mortality in the overall cohort.

Variables	Patient Number (%)		Univariate Analysis		Multivariate Analysis	
	Death *n* = 56	Survival *n* = 885	Odds Ratio (95% CI)	*p* Value	Odds Ratio (95%CI)	*p* Value
Gender, male	37 (66.1)	359 (40.6)	2.85 (1.62–5.04)	<0.001	NS	NS
Pitt bacteremia score						
≥4 at onset	30 (53.6)	117 (13.2)	7.57 (4.33–13.26)	<0.001	2.59 (1.34–5.03)	0.005
=0 ay day 3	11 (19.6)	690 (78.0)	0.07 (0.04–0.14)	<0.001	0.21 (0.11–0.41)	<0.001
Ultimately and rapidly fatal comorbidities (McCabe classification)	31 (55.4)	165 (18.6)	5.41 (3.11–9.41)	<0.001	3.20 (1.62–6.33)	0.001
Comorbidities						
Malignancies	30 (53.6)	221 (25.0)	3.47 (2.01–5.99)	<0.001	1.98 (1.02–3.86)	0.04
Hypertension	18 (32.1)	444 (50.2)	0.47 (0.26–0.84)	0.009	NS	NS
Liver cirrhosis	18 (32.1)	108 (12.2)	3.41 (1.88–6.18)	<0.001	2.15 (1.08–4.27)	0.03
Sources of bacteremia						
Pneumonia	22 (39.3)	69 (7.8)	7.65 (4.24–13.80)	<0.001	3.19 (1.64–6.23)	0.001
Urinary tract infections	10 (17.9)	466 (52.7)	0.20 (0.10–0.39)	<0.001	0.32 (0.16–0.52)	0.001

NS = No significance (after processing the stepwise and backward multivariate regression).
